# iNOS Activity Modulates Inflammation, Angiogenesis, and Tissue Fibrosis in Polyether-Polyurethane Synthetic Implants

**DOI:** 10.1155/2015/138461

**Published:** 2015-05-27

**Authors:** Puebla Cassini-Vieira, Fernanda Assis Araújo, Filipi Leles da Costa Dias, Remo Castro Russo, Silvia Passos Andrade, Mauro Martins Teixeira, Luciola Silva Barcelos

**Affiliations:** ^1^Laboratório de Angiogênese e Células-Tronco, Departamento de Fisiologia e Biofísica, Universidade Federal de Minas Gerais, 31270-901 Belo Horizonte, MG, Brazil; ^2^Laboratório de Imunofarmacologia, Departamento de Bioquímica e Imunologia, Universidade Federal de Minas Gerais, 31270-901 Belo Horizonte, MG, Brazil; ^3^Laboratório de Angiogênese, Área de Ciências Fisiológicas-ARFIS, Universidade Federal de Uberlândia, 38405-320 Uberlândia, MG, Brazil; ^4^Laboratório de Imunologia e Mecânica Pulmonar, Departamento de Fisiologia e Biofísica, 31270-901 Belo Horizonte, MG, Brazil

## Abstract

There is considerable interest in implantation techniques and scaffolds for tissue engineering and, for safety and biocompatibility reasons, inflammation, angiogenesis, and fibrosis need to be determined. The contribution of inducible nitric oxide synthase (iNOS) in the regulation of the foreign body reaction induced by subcutaneous implantation of a synthetic matrix was never investigated. Here, we examined the role of iNOS in angiogenesis, inflammation, and collagen deposition induced by polyether-polyurethane synthetic implants, using mice with targeted disruption of the iNOS gene (iNOS^−/−^) and wild-type (WT) mice. The hemoglobin content and number of vessels were decreased in the implants of iNOS^−/−^ mice compared to WT mice 14 days after implantation. VEGF levels were also reduced in the implants of iNOS^−/−^ mice. In contrast, the iNOS^−/−^ implants exhibited an increased neutrophil and macrophage infiltration. However, no alterations were observed in levels of CXCL1 and CCL2, chemokines related to neutrophil and macrophage migration, respectively. Furthermore, the implants of iNOS^−/−^ mice showed boosted collagen deposition. These data suggest that iNOS activity controls inflammation, angiogenesis, and fibrogenesis in polyether-polyurethane synthetic implants and that lack of iNOS expression increases foreign body reaction to implants in mice.

## 1. Introduction

The implantation of polyether-polyurethane synthetic matrix has been used as a framework to induce fibrovascular tissue growth that has similarities with tissue formed in the physiological and pathological processes where angiogenesis, inflammation, and fibrogenesis are important concurrent processes. This model provides a chronically inflamed environment that mimics the ones that occur after mechanical tissue injuries such as in balloon angioplasty insertion, as well as during wound healing, inflammatory diseases, and foreign body reaction [1]. Indeed, at present, there is considerable interest in implantation techniques and scaffolds for tissue engineering and, for safety and biocompatibility reasons, inflammation, angiogenesis, and fibrosis need to be determined, as the success of the procedure is conditioned by an adequate inflammatory angiogenesis response to the device as well as by its degree of encapsulation [1, 2].

One of the main inflammatory mediators involved in both inflammation and angiogenesis is the nitric oxide (NO). NO can be synthesized by three different isoforms of NO synthase: neuronal (nNOS), endothelial (eNOS), and inducible (iNOS) synthases [3]. NO production due to cytokine-induced expression of inducible nitric oxide synthase (iNOS) is largely involved in the pathophysiology of inflammation [4–6]. Studies conducted in iNOS-deficient mice and/or under iNOS inhibition have provided evidence that inducible NO governs a broad spectrum of processes, such as recruitment and adhesion of leukocyte [7, 8], inflammatory diseases [9, 10], wound healing [11, 12], ischemia [13–15], and tumor-induced angiogenesis [16]. In addition, NO-releasing compounds have proved effective in attenuating foreign body reaction to subcutaneous implant [17, 18]. While these studies have been decisive in demonstrating the potential activity of NO in minimizing the adverse foreign body reaction, we found no study that investigated the role of endogenous iNOS in the regulation of the foreign body reaction induced by subcutaneous implantation of synthetic matrix in mice.

We hypothesized that iNOS might modulate inflammatory angiogenesis in synthetic implants. Therefore, our aim was to study the effects of iNOS deletion on different features of the foreign body response induced by polyether-polyurethane implants in terms of inflammation, neovascularization, and fibrogenesis in the synthetic matrix. We report that the deletion of iNOS was able to modulate critical features of inflammation, neovascularization, and collagen deposition on the fibrovascular tissue induced by sponge implants in mice as the absence of this enzyme leads to reduced angiogenesis and exacerbated inflammation and fibrosis in the synthetic implants.

## 2. Material and Methods

### 2.1. Animals

All animal care and experimental procedures complied with the guidelines established by our local Institutional Animal Welfare Committee. Efforts were made to avoid all unnecessary distress to animals. Male C57BL/6 mice aged 7-8 weeks (20–25 g body weight) with genetic deletion of iNOS (iNOS^−/−^) and wild-type (WT) mice were provided by Dr. Leda Quércia Vieira (Department of Biochemistry and Immunology at the Institute of Biological Sciences, Federal University of Minas Gerais, Brazil). The animals were housed individually and provided with chow pellets and water* ad libitum*. The light/dark cycle was 12:12 h with lights on at 7:00 am and lights off at 7:00 pm.

### 2.2. Sponge Discs Implantation

Polyether-polyurethane sponge (Vitafoam Ltd., Manchester, UK) was used as the implanted material, as described previously [2, 19, 20]. The implants were in the shape of discs, 5 mm thick × 8 mm diameter. They were soaked overnight in 70% v/v ethanol and sterilized by boiling in distilled water for 15 minutes before implantation. The animals were anaesthetized with a mixture of ketamine 100 mg/kg and xylazine 10 mg/kg and the dorsal hair was shaved and the exposed skin wiped with 70% ethanol. The sponge discs were aseptically implanted into a subcutaneous pouch, which had been made with curved artery forceps through a 1-cm long dorsal midline incision. Postoperatively, the animals were monitored for any signs of infection at the surgical site, discomfort, or distress; any animals showing such signs were promptly euthanized with anesthetic excess. The implants were evaluated 14 days after implantation to assess vascularization (hemoglobin content, cytokines levels, and histological analysis), inflammatory markers (MPO and NAG activities and cytokines levels), and collagen deposition (Picrosirius-red staining).

### 2.3. Hemoglobin Extraction and Measurement

The extent of vascularization of the sponge implants was assessed by the amount of hemoglobin (Hb) detected in the tissue using the Drabkin method [19, 20]. At 14 days after implantation, the animals were euthanized with an excess of anesthetic and the sponge implants were carefully removed, dissected, cleared of any adherent tissue, and weighed. Each implant was homogenized (Tekmar TR-10, OH) in 2 mL of Drabkin reagent (Labtest, São Paulo, Brazil) and centrifuged at 12000 ×g for 20 min. The supernatants were filtered through a 0.22-*μ*m Millipore filter. The hemoglobin concentration in the samples was determined spectrophotometrically by measuring absorbance at 540 nm using an ELISA plate reader and comparing it against a standard hemoglobin curve. Hemoglobin content in the implant was expressed as *μ*g of Hb per mg wet tissue.

### 2.4. ELISA for Cytokines/Chemokines

The supernatants from centrifugation of sponge homogenates (see hemoglobin measurement method) were used to examine the levels of VEGF, CXCL1/KC, CCL2/MCP-1, TNF-*α*, IL-10, and IFN-*γ* produced in sponge implants by ELISA. The assays were performed using kits from R&D Systems and according to the manufacturer's instructions. Standards were 0.5-log_10_ dilutions of recombinant murine cytokines from 7.5 pg mL^−1^ to 1000 pg mL^−1^. The threshold of sensitivity for each cytokine/chemokine was 7.5 pg/mL. The results were expressed as pg cytokine per mg wet tissue.

### 2.5. Quantification of Neutrophil or Macrophage Tissue Accumulation

Pellets from centrifugation of sponge homogenates (see hemoglobin measurement method) were divided into two portions and suspended with different buffers specific for measurement of myeloperoxidase (MPO) or N-acetyl-*β*-D-glucosaminidase (NAG) activities used as neutrophil and macrophage accumulation indexes, respectively, as described previously [20].

### 2.6. Histological Analysis

The sponge implants from a separate group of mice were carefully excised, dissected free of adherent tissue, and fixed in formalin (10% w/v in isotonic saline). Sections (5 *μ*m) were stained with hematoxylin and eosin (H&E) or Picrosirius-red and processed for light-microscopic studies. To perform a morphometric analysis of blood vessels, cross section images obtained from 15 sequential fields (8533 *μ*m^2^) from each implant were captured with a plan apochromatic objective (40x) in light microscopy (final magnification = 400x). A countable vessel was defined as a structure with a lumen whether or not it contained red blood cell [1]. For collagen analysis, images were obtained from 10 fields (area = 343,592 *μ*m^2^) at 20x (final magnification = 200x) under polarized light (Olympus). The images were digitized through an Olympus BX43 with an Olympus Q-color 5 microcamera and morphometric analyses were performed on digital images using ImageProPlus 7.0 Software. A single observer blinded to the condition and treatment performed the analysis. Additionally, to standardize the image analysis, the fields were photographed on the same day to avoid any variability associated with the light source.

### 2.7. Statistical Analysis

The results were expressed as mean ± SEM. Statistical comparisons between two groups of mice were carried out using Student's *t*-test for unpaired data. Differences between means were considered significant when *p* values were *p* < 0.05. The statistical analysis was performed using GraphPad Prism 6.0.

## 3. Results

### 3.1. Angiogenesis Is Impaired in Synthetic Implants from iNOS-Deficient Mice

**Figure 1:**
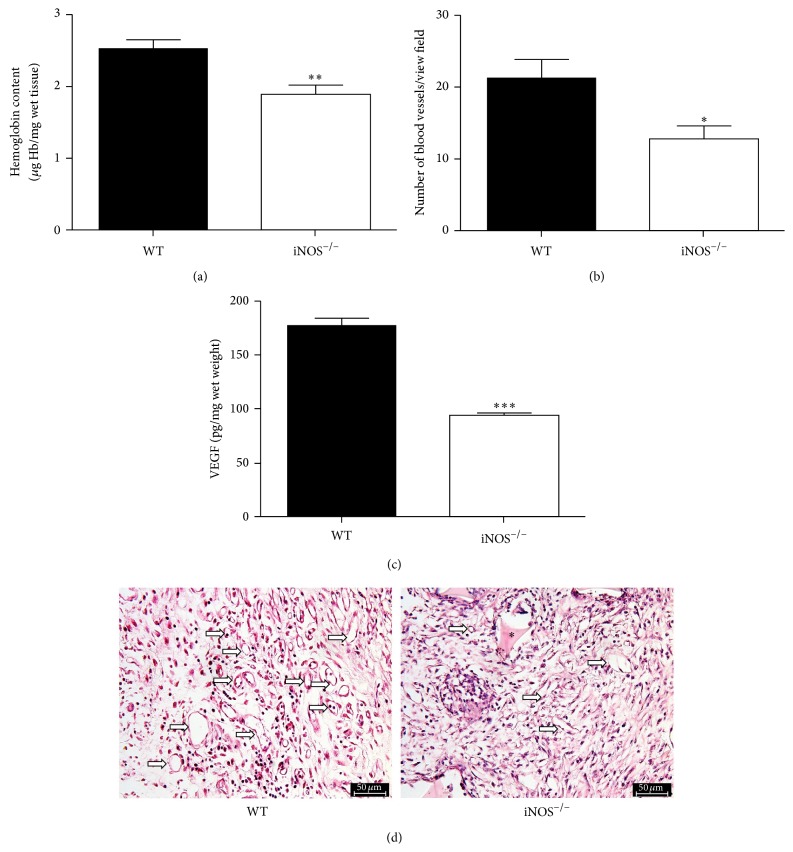
Impaired angiogenesis in implants from iNOS^−/−^ mice. Hemoglobin content (a). Blood vessels density (b). VEGF levels (c). Representative photomicrographs of H&E-stained histological sections (d). The sponges were collected for analyses at day 14 after implantation. Values were represented as means (± SEM) from groups of 8 animals each. ^*∗∗*^ *p* < 0.01, ^*∗∗∗*^ *p* < 0.001 versus WT group (Student's *t*-test). Bar 50 *μ*m. Asterisks indicate the synthetic matrix. White arrows indicate blood vessels.

The sponge matrix was well tolerated by animals of both wild-type and knockout groups. No signs of infection or rejection were observed in the implant location during the 14-day period of the experiment. Angiogenesis was assessed by evaluating implants hemoglobin content and blood vessels counting in H&E-stained tissue sections, besides VEGF levels by ELISA. We observed reduced hemoglobin content (Figure 1(a)) and blood vessels count (Figures 1(b) and 1(d)) in the fibrovascular tissue at day 14 after sponge implantation in iNOS^−/−^ mice when compared to the WT counterparts. In WT mice, the hemoglobin content (*μ*g/mg wet tissue) was 2.52 ± 0.12 and, in iNOS^−/−^ mice, it fell to 1.88 ± 0.13 (*p* < 0.01). Corroborating these data, the measurement of the proangiogenic cytokine VEGF was also decreased in sponges from iNOS^−/−^ mice, as shown in Figure 1(c). Altogether, these data suggest that sponge-induced angiogenesis is impaired in the absence of iNOS activity.

### 3.2. Leukocyte Infiltration Is Enhanced in Implants from iNOS-Deficient Mice

**Figure 2:**
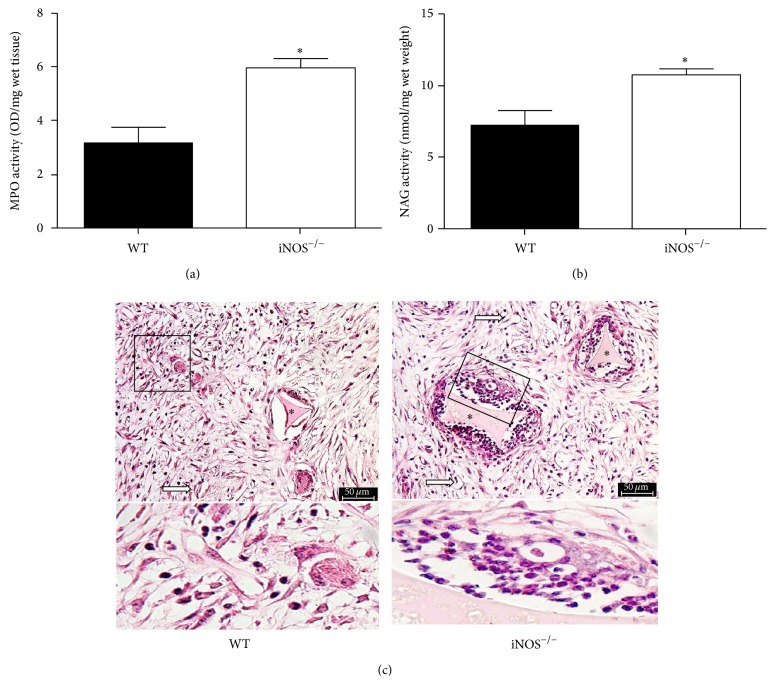
Increased leukocyte accumulation in implants from iNOS^−/−^ mice. The contents of neutrophils (a) and macrophages (b) recruited to the implant were determined by myeloperoxidase (MPO) and N-acetyl-*β*-D-glucosaminidase (NAG) activities, respectively. Representative photomicrographs of H&E-stained histological sections (c). The sponges were collected at day 14 after implantation. Values were represented as means (± SEM) from groups of 8 animals each. ^*∗*^ *p* < 0.05 versus WT group (Student's *t*-test). Bar 50 *μ*m. Asterisks indicate the synthetic matrix. White arrows indicate blood vessels.

The inflammatory component was determined by estimating the neutrophil and macrophage accumulation into the implants through assaying MPO and NAG enzyme activities, respectively, as well as measuring levels of cytokines (CXCL1, CCL2, TNF-*α*, IL-10, and IFN-*γ*) in the implants. Oppositely to the reduced formation of new blood vessels in the newly formed fibrovascular tissue, we observed an enhancement in the leukocyte accumulation into implants of iNOS^−/−^ mice when compared to WT, as assayed by MPO (Figure 2(a), *p* < 0.05) and NAG activities (Figure 2(b), *p* < 0.05), suggesting increased infiltration of neutrophils and macrophages, respectively. The histopathology confirmed the increased leukocyte infiltration in implants from iNOS^−/−^ mice compared to WT mice (Figure 2(c)).

**Figure 3:**
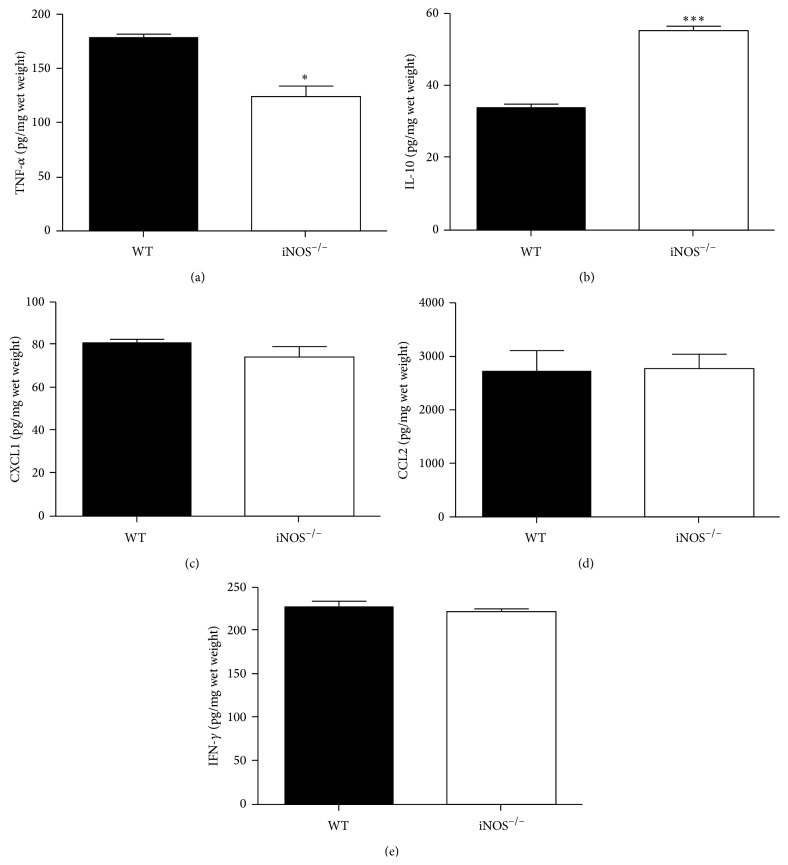
Modulation of cytokines, but not chemokines production, in implants from iNOS^−/−^ mice. TNF-*α* (a), IL-10 (b), CXCL1/KC (c), CCL2/MCP-1 (d), and IFN-*γ* (e) levels were evaluated by sandwich-type ELISA. The sponges were collected at day 14 after implantation. Values were represented as means (±SEM) from groups of 8 animals each. ^*∗*^ *p* < 0.05, ^*∗∗*^ *p* < 0.01, and ^*∗∗∗*^ *p* < 0.001 versus WT group (Student's *t*-test).

Interestingly, levels of the proinflammatory cytokine TNF-*α* (Figure 3(a), *p* < 0.05) were decreased, while levels of the anti-inflammatory cytokine IL-10 (Figure 3(b), *p* < 0.001) were increased in sponges from iNOS^−/−^ mice when compared with WT mice. However, no differences were observed in CXCL1 (Figure 3(c)), CCL2 (Figure 3(d)), or IFN-*γ* (Figure 3(e)) levels between both groups.

### 3.3. Collagen Deposition Is Increased in Implants from iNOS-Deficient Mice

**Figure 4:**
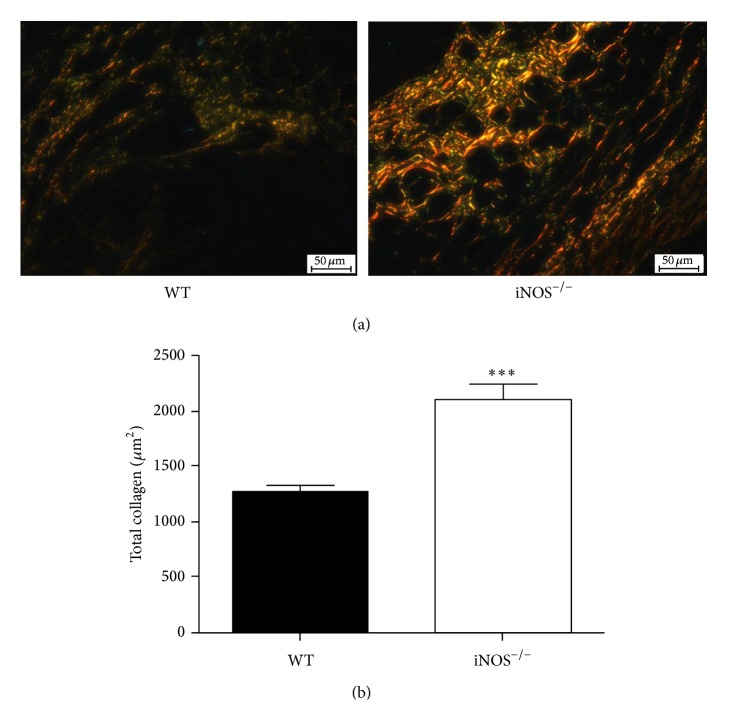
Enhanced collagen deposition in implants from iNOS^−/−^ mice. Representative photomicrographs of* Picrosirius*-*red*-stained sections (a) for collagen assessment under polarized light microscopy. Morphometric analysis of collagen content (b). The sponges were collected at day 14 after implantation. Values were represented as means (±SEM) from groups of 8 animals each. ^*∗∗∗*^ *p* < 0.001 versus WT group (Student's *t*-test). Bar 50 *μ*m.

Another important component of the fibrovascular tissue is the collagen deposition. Here, we estimated the content of collagen present in the implants by calculating the total area occupied by collagen (*μ*m^2^) in Picrosirius-red-stained histological sections evaluated under polarized light (Figure 4(a)). As shown in Figure 4(b), there was a significant increase in collagen deposition in implants from iNOS^−/−^ mice when compared with WT mice (*p* < 0.001), suggesting iNOS activity plays a role in adjusting collagen deposition during the inflammatory angiogenesis process induced by an implanted device.

## 4. Discussion

Nitric oxide (NO) is a diffusible gas involved in a vast number of biological processes that cover the cardiovascular, immune, and neural systems. Its production can be achieved via three known different NO synthase (NOS) enzymes isoforms: nNOS (neuronal; NOS1), iNOS (inducible; NOS2), and eNOS (endothelial; NOS3). Different from neuronal and endothelial isoforms, iNOS is regulated mainly on transcriptional level and is independent of intracellular calcium concentration [6]. Besides its important physiological roles in cardiovascular functions, especially in endothelium-dependent vasorelaxation, antiplatelet aggregation, and leukocyte adhesion, NO is a key mediator of inflammation ensuring antimicrobial and immunoregulatory functions as well as modulating by leukocyte recruitment [21]. The expression of iNOS, specifically, is involved in many inflammatory and neoplastic conditions [16]. More recently, the role of iNOS-induced NO during ischemia was also demonstrated [22]. All these physiopathological situations depend on new blood vessel formation to perpetuate or even cease.

In addition, considering the interest in implantation techniques and scaffolds for tissue engineering, it is worth mentioning that, as demonstrated by our group, levels of NO in polyether-polyurethane synthetic sponge implants are modulated by diverse angiogenesis-modifying treatments without, however, a definite correlation with inflammatory cell accumulation [23, 24]. Moreover, iNOS activity seems to be crucial for adequate response to polypropylene implant integration in the peritoneum [25], although not necessary for the collagen deposition in synthetic PVA (polyvinyl-alcohol) sponges [26]. Nevertheless, the contribution of the iNOS isoform to the formation of a fibrovascular tissue in the polyether-polyurethane sponge implants was never investigated. Here, we studied the role of iNOS in different features of the foreign body response induced by that matrix implantation into the subcutaneous compartment of mice. Such implants are known to be infiltrated by a number of cells such as inflammatory cells, endothelial cells, and fibroblasts [19].

We demonstrated that iNOS expression may modulate the major concurrent components of the newly formed fibrovascular tissue (angiogenesis, inflammation, and collagen deposition) induced by sponge implantation. In the first set of results, we observed that angiogenesis and VEGF levels are reduced in the implants from iNOS^−/−^ mice when compared to WT animals. Those data are in agreement with various reports in different* in vitro* and* in vivo* systems that demonstrated inhibition of iNOS results in decreased VEGF levels and consequently impaired angiogenesis [27–29]. Indeed, the NO production by iNOS may, for instance, contribute to the release of VEGF [30], a growth factor known to be important to stimulate endothelial cell migration and the angiogenic process* in vivo* [31, 32]. VEGF, in turn, may stimulate eNOS activity resulting in further release of NO [26, 33, 34]. Therefore, our results suggest that impaired angiogenesis in the sponge implants from iNOS^−/−^ mice may be attributed, at least partially, to the reduced NO-induced VEGF levels.

Angiogenesis and inflammation are interconnected processes in many pathophysiological conditions as well as in response to synthetic devices [35]. Here, we measured several markers of inflammation (MPO and NAG activities and TNF-*α*, CXCL1/KC, CCL2/MCP-1, IFN-*γ*, and IL-10 levels) in 14-day-old implants from WT and iNOS^−/−^ animals. We observed that iNOS deficiency resulted in a prolonged inflammatory cell infiltration into the implants with both neutrophils and macrophages content being increased in sponges from iNOS^−/−^ when compared to WT mice. However, no differences were observed in the levels of the respective chemoattractants CXCL1 or CCL2. In effect, there is a vast literature demonstrating that NO is a vascular protective molecule that prevents leukocyte adhesion to the endothelium and trafficking [36]. Likewise, there is evidence that iNOS-derived NO may modulate leukocyte and platelet functions that are involved in leukocyte recruitment to inflammatory site [37, 38]. Hickey et al. [39], using a LPS* in vitro* model, showed that iNOS-derived nitric oxide was able to reduce the adhesion of leukocytes under hydrodynamic flow conditions, providing a plausible explanation for the enhanced leucocyte recruitment observed* in vivo* in iNOS^−/−^ mice. Therefore, it is reasonable to speculate that the signaling pathways of the inflammatory chemokines CXCL1 and CCL2 are preserved at some extension in the implants from iNOS^−/−^ mice and that the increased accumulation of inflammatory cells in the sponge from these animals may be attributed to the lack of iNOS-derived NO production, but not increase in the chemoattractant production.

Intriguingly, we found reduced levels of the proinflammatory cytokine TNF-*α* in the implants from iNOS^−/−^ mice but increased levels of the anti-inflammatory cytokine IL-10, when compared with WT mice. In accordance, decreased production of proinflammatory cytokines in iNOS^−/−^ mice has been shown [40, 41]. In addition, a compensatory overproduction of IL-10 may be observed in mice lacking the gene for the enzyme iNOS [42, 43]. Of note, IL-10 is associated with an antiangiogenic effect by its ability to decrease VEGF levels [15] which, in turn, could partially help to explain the reduced levels of VEGF in the implants.

The collagen deposition during tissue remodeling is regulated by complex interactions of pro- and antifibrogenic proteins within the inflammatory tissue [44–46]. There are some pieces of evidence in the literature regarding the NO regulation of collagen deposition as, for example, increased collagen deposition observed in excisional wounds in iNOS^−/−^ mice [12]. In addition, neutrophils and macrophages are well known to be involved in inflammation-induced fibrosis [47]. Therefore, the accumulation of inflammatory cells into the implants of iNOS-deficient mice could be one possible explanation of why there is an exaggerated fibrotic response in the implants from those animals. In fact, in the absence of iNOS activity, macrophages may acquire a nonclassical activated phenotype that may have profibrotic characteristics [48, 49] and then may account for the increased collagen deposition in the implants from iNOS-deficient mice. In addition, it is worth mentioning that although IL-10 is understood as an antifibrotic cytokine, it has been suggested that IL-10 cooperates with Th1 cytokines, such as IFN-*γ* (and even TNF-*α*), to suppress collagen deposition [50, 51]. Interestingly, here, there were no changes in IFN-*γ* levels, and TNF-*α* levels were even decreased, indicating a possible lack of the regulatory pathway for collagen deposition and, consequently, a supportive microenvironment for increasing fibrogenesis in the implants is endorsed in the absence of iNOS activity. Altogether, our data suggest that iNOS expression helps in controlling fibrosis in the sponge implants.

Overall, the absence of iNOS leads to increased foreign body reaction to implants in mice, observed as reduced angiogenesis and exacerbated inflammation and fibrosis in the synthetic implants. Therefore, the present work suggests a potential role for iNOS as a regulator of inflammatory angiogenesis during the fibrovascular tissue formation in sponge implants. These observations may provide relevant information for future insights in therapeutic strategies for conditions where inflammation and angiogenesis are associated as well as for the development of scaffolds for tissue engineering.

## Abbreviations

CCL2: Chemokine (C-C motif) ligand 2
CCR 2: C-C chemokine receptor type 2
CXCL1: Chemokine (C-X-C motif) ligand 1
ELISA: Enzyme Linked Immunosorbent Assay
HTAB: Hexadecyltrimethylammonium bromide
IFN-*γ*: Interferon gamma
IL-10: Interleukin 10
iNOS: Inducible nitric oxide synthase
KO: Knockout mice
MPO: Myeloperoxidase
NAG: N-Acetyl-*β*-D-glucosaminidase
NO: Nitric oxide
NOS: Nitric oxide synthase
OD: Optic density
SEM: Standard error of the mean
TNF-*α*: Tumor necrosis factor-*α*
VEGF: Vascular endothelial growth factor
WT: Wild-type.
